# *Staphylococcus epidermidis* role in the skin microenvironment

**DOI:** 10.1111/jcmm.14415

**Published:** 2019-07-06

**Authors:** Caroline Leonel, Isadora F. G. Sena, Walison N. Silva, Pedro H. D. M. Prazeres, Gabriel R. Fernandes, Pamela Mancha Agresti, Mariana Martins Drumond, Akiva Mintz, Vasco A. C. Azevedo, Alexander Birbrair

**Affiliations:** ^1^ Departamento de Patologia Universidade Federal de Minas Gerais Belo Horizonte Brasil; ^2^ Oswaldo Cruz Foundation, René Rachou Research Center Belo Horizonte Brasil; ^3^ Departamento de Biologia Geral Universidade Federal de Minas Gerais Belo Horizonte Brasil; ^4^ Department of Radiology Columbia University Medical Center New York New York

**Keywords:** microbiota, microenvironment, skin, wound healing

## Abstract

Wound healing is a complex dynamic physiological process in response to cutaneous destructive stimuli that aims to restore the cutaneous’ barrier role. Deciphering the underlying mechanistic details that contribute to wound healing will create novel therapeutic strategies for skin repair. Recently, by using state‐of‐the‐art technologies, it was revealed that the cutaneous microbiota interact with skin immune cells. Strikingly, commensal *Staphylococcus epidermidis*‐induced CD8+ T cells induce re‐epithelization of the skin after injury, accelerating wound closure. From a drug development perspective, the microbiota may provide new therapeutic candidate molecules to accelerate skin healing. Here, we summarize and evaluate recent advances in the understanding of the microbiota in the skin microenvironment.

## 1 INTRODUCTION

The skin is the largest organ in surface area, covering approximately two square metres, and maintaining the body integrity. It grants interaction with our environment, concurrently protecting from its multiple possible physical, mechanical, chemical and biological assaults. Additionally, the skin plays an essential role in maintaining the human body “healthy”, preventing external pathogens from entering our organism, balancing body temperature, transmitting sensations, and averting loss of vital internal fluids. The skin comprises an outermost layer, the epidermis, a subjacent connective tissue, the dermis, and the subcutaneous tissue. Each region is made of several components, including keratinocytes, melanocytes, and Langerhans cells in the epidermis, immune cells, fibroblasts, nerve endings, pericytes, and endothelial cells in the dermis, and adipocytes in the subcutaneous tissue.^1^, ^2^ Changes in the cutaneous constituents of the skin barrier may lead to pathologic conditions, including sterile skin inflammation, allergic sensitization, skin infections, cutaneous tumour development, or delay in skin healing.^3^

Cutaneous wounds may be caused upon perturbation of the cutaneous barrier by burns, irradiation, traumatic injury, ulcers, chronic inflammation or other insults. If the damage to the skin is not properly fixed, it may lead to infection, pain, and unwanted sequelae, including scar formation. Skin wound healing is a dynamic complex process of reestablishing the shielding barrier that protects our body from the environment, subdivided overlapping steps: inflammation, proliferation and tissue remodelling. A coordinated balance between distinct cell types, molecules released by those cells, innervations, extracellular matrix proteins, and other components of the cutaneous microenvironment is essential for normal skin wound repair.^4^ The lack of a detailed understanding about the multiple factors by which cutaneous healing may be affected restricts the design of effective treatments that would accelerate and improve skin restoration. Deciphering all contributors to skin wound healing will have positive effects on patients’ lives.

**Figure 1:**
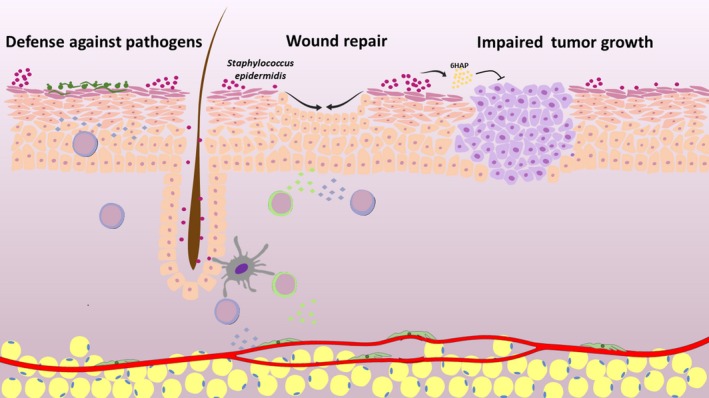
*Staphylococcus epidermidis* induce wound healing and tumour regression in the skin. *Staphylococcus epidermidis* are the predominant gram‐positive commensal bacteria that colonize normal skin. The studies of Linehan et al (2018) and Nakarsuji et al (2018) now suggest novel roles for *Staphylococcus epidermidis* in the skin microenvironment.^7^, ^8^ *Staphylococcus epidermidis* promote wound healing via accumulation of CD8+ T lymphocytes in the skin, and suppress cutaneous tumour formation via 6‐N‐hydroxyaminopurine secretion. Future studies will reveal in detail the cellular and molecular mechanisms involved in the interactions between the cutaneous microbiota and several components of the skin microenvironment

The skin, although being a physical barrier for foreign pathogenic microbes, is colonized by diverse commensal bacteria which may be beneficial for cutaneous homeostasis. For instance, these bacteria provide important nutrients, affecting cellular metabolism and strengthen the immune system.^5^ Therefore, it is not surprising that the loss of protective bacteria exacerbates inflammatory skin diseases.^6^ How signals produced by commensal bacteria are recognized by the host cells, and how these molecules affect the skin microenvironment during wound healing remain largely unknown. Now, in a recent article in *Cell*, Linehan et al investigated how the cutaneous microbiota interacts with the skin immune cells.^7^ The authors revealed, by using state‐of‐the‐art techniques, that bacteria‐derived peptides are direct mediators of the cross‐talk between cutaneous bacteria and the host cutaneous cells (Figure 1). Linehan and colleagues reported that *Staphylococcus epidermidis* induces accumulation of CD8+ T lymphocytes in the skin via antigen presentation by dendritic cells.^7^ Interestingly, the same isolates of *Staphylococcus epidermidis* are enriched in the skin of healthy patients in comparison with adult humans with skin disease. The authors showed, by analysis of the *Staphylococcus epidermidis* genome in combination with in vitro experiments, that major histocompatibility complex Ib (MHCIb) on dendritic cells present N‐formyl methionine peptides, secreted by *Staphylococcus epidermidis*, to CD8+ T lymphocytes.^7^ Moreover, Linehan et al performed RNA sequencing of CD8+ T lymphocytes induced by topical association with *Staphylococcus epidermidis*. The analysis of global transcriptome signature unveiled that CD8+ T lymphocytes up‐regulate multiple genes associated with immune regulation, and tissue repair‐related genes. Additionally, the authors examined the effect of *Staphylococcus epidermidis* previous exposure on wound healing by using punch biopsy method to induce skin injury. Strikingly, *Staphylococcus epidermidis*‐induced CD8+ T cells promoted improved re‐epithelization of the affected skin, and accelerating wound repair.^7^ Here, we discuss these findings, and evaluate recent advances in our understanding of how the cutaneous microbiota affects the skin microenvironment.

## 2 PERSPECTIVES/FUTURE DIRECTIONS

### 2.1 Presentation of *Staphylococcus epidermidis* antigens in the skin

Recent studies have provided evidence that in addition to dendritic cells, other cells present in the skin, like B cells, macrophages, epidermal Langerhans cells, keratinocytes, and neutrophils, may function as antigen‐presenting cells.^9^, ^10^, ^11^, ^12^ For instance, staphylococcal lipoteichoic acid inhibits inflammation, by acting on keratinocytes via toll‐like receptor 3.^13^ Linehan et al suggest that *Staphylococcus epidermidis* antigens are presented by dendritic cells via MHCIb.^7^ The exact cellular and molecular mechanisms involved in *Staphylococcus epidermidis*–derived peptides presentation in vivo are yet not completely clear, and will need to be revealed in future studies. Transgenic mice have been widely applied to study the roles of multiple cell populations within distinct tissue‐microenvironments.^14^, ^15^ The ability, not only to eliminate cells, but also to delete single genes in individual cellular populations in adult mice has allowed us to answer key questions regarding the roles of specific proteins in different cell subsets in the regulation of several physiologic processes. The major histocompatibility complexes have not been conditionally deleted from cutaneous dendritic cells or other possible antigen‐presenting cells, so there is no direct evidence whether dendritic cells are the only/main functionally important antigen‐presenting cells in the interaction with *Staphylococcus epidermidis*. The generation of MHCIb‐floxed mice to be crossed with dendritic cell‐specific inducible CreER driver will allow us to specifically delete this molecule in vivo. In addition to studies in genetic mouse models, transcriptomic and single dendritic cell analysis represent fundamental tools that will help us understand the role of dendritic cells in the cross‐talk with the cutaneous microbiota. Moreover, it will be interesting to examine whether those cells are also important for presentation of antigens derived from other constituents of the cutaneous commensal microbiota. Additionally, it will be import to evaluate whether other cells besides CD8+ T cells are activated by *Staphylococcus epidermidis*‐derived N‐formyl methionine peptides.

In homeostatic conditions, the human cutaneous microenvironment contains multiple resident T cells.^16^ Probably, these cells result from antigen presentation of the cutaneous microbiota. Their exact roles in the skin remain unclear. Linehan and collaborators have shown that commensal‐specific T cells also promote wound repair.^7^ The influence of the skin microbiome commensal members on the immune system should be further explored in future studies.

### 2.2 Influence of microbiota on the skin microenvironment during wound healing

The skin is composed by a complex microenvironment which contains several constituents, in addition to dendritic cells and CD8+ T cells: other immune cells, various types of stromal cells, innervations, stem cells, Schwann cells, and extracellular matrix proteins.^14^, ^17^, ^18^, ^19^, ^20^, ^21^, ^22^, ^23^, ^24^, ^25^, ^26^, ^27^, ^28^, ^29^, ^30^, ^31^, ^32^, ^33^, ^34^, ^35^, ^36^, ^37^, ^38^, ^39^, ^40^, ^41^, ^42^, ^43^, ^44^, ^45^, ^46^, ^47^, ^48^, ^49^, ^50^, ^51^, ^52^, ^53^, ^54^, ^55^, ^56^, ^57^ This complex mixture of cells cooperate to perform the necessary functions for the skin healing, and the interplay between these distinct components of the cutaneous microenvironment will define the success of skin repair.^58^, ^59^ In addition, to this complexity, now we introduce the microbes as important players in this niche, which may affect wound healing. Interestingly, a recent study has suggested that absence of commensal microbiota in the skin accelerates wound closure.^60^ In combination with the positive effects exerted by *Staphylococcus epidermidis* on wound healing demonstrated by Linehan et al (2018), these data indicated that some commensal bacteria may affect injury repair negatively in the skin, which effects are nullified by *Staphylococcus epidermidis*.

However, in another study, mice orally treated with vancomycin exhibited skin microbiota dysbiosis and delayed wound closure.^61^ These experiments demonstrated the complex role of skin microbiome in wound repair. In addition to the work of Linehan et al, other studies have suggested a beneficial relationship between the microbiome and wound healing.^13^ The wound healing is a dynamic process associated with global variance in the skin microbiome. In contrast to these findings, the stability of wound microbiota, even though temporary, is associated with poor healing.^62^ Therefore, understanding the skin microbiome dysbiosis will be important in association with analysing isolated pathogens in the wounds.

Deciphering the individual and combinatorial signals derived from different cutaneous bacteria that influence skin healing will help develop novel treatments. Thus, what is the cross‐talk between distinct components of the cutaneous commensal microbiota involved in skin healing and other cutaneous microenvironment cells remains to be revealed. For instance, future studies are required to evaluate the importance of *Staphylococcus epidermidis*’ interactions with other immune cells and stem cells in skin healing. Moreover, it remains unclear what is the bacterial load of* Staphylococcus epidermidis* necessary for their roles in the skin microenvironment. Also, future studies should explore whether the biofilm derived from *Staphylococcus epidermidis* hurts wound healing. Additionally, as the wound may have several types of bacteria, it will be interesting to explore how other bacteria influence T cells, and whether they cross‐talk with* Staphylococcus epidermidis* in the skin. Further insights into the molecular and cellular mechanisms participating in wound healing will implicate in our understanding of skin homeostasis and response to injury.

### 2.3 Heterogeneity of skin micrbiota

Cutaneous microbial communities located at different parts of the body are heterogeneous.^63^ This may be due to complex chemical, biological, and physical composition across distinct skin regions. For instance, cutaneous regions as the sole of the foot and the index finger harbour microbiomes that differ between each other.^64^ Also, *Cutibacterium* predominate in the face,^65^ while *β‐Proteobacteria* and *Corynebacterium* are more abundant in the elbows and knees.^63^ Interestingly, *Staphylococcus epidermidis* differ in the strains that colonize among distinct body sites of the same patient.^66^ As it has been shown recently, strains from the same species may differ in their biologic effects on the host.^67^ Thus, strain‐level differences should be considered in the skin microbiota. As skin regions also vary in regard to their variety and density of hair follicles and glands, future studies should explore whether the role of specific strains of *Staphylococcus epidermidis* on distinct regions of the skin varies in CD8+ T cells activation. If so, it remains to be determined which *Staphylococcus epidermidis* strain can be used for clinical applications.

The skin is subjected to cumulative and sequential alterations with the passage of time.^68^ Few studies have been conducted on the effect of ageing in skin microbiomes.^69^, ^70^, ^71^, ^72^, ^73^, ^74^, ^75^ Futures studies should explore further whether/how changes with ageing in skin microbiomes affect cutaneous physiological properties as well.

### 2.4 Role of *Staphylococcus epidermidis* in the skin tumour microenvironment

There have been a long‐standing link between cancer and wound development, with tumours described as wounds that do not heal.^76^ Recently, biological processes that arise during normal wound repair have been associated to tumorigenesis.^77^, ^78^, ^79^, ^80^, ^81^, ^82^ However, little is known about the mechanistic details and the role of the commensal microbiota in these phenomena. Now, in a recent study in *Science Advances*, Nakatsuji and colleagues show that the microbiome protects against cutaneous tumour development.^8^ The authors revealed that *Staphylococcus epidermidis* produces a molecule with anti‐tumoural activity (6‐N‐hydroxyaminopurine). Interestingly, 6‐N‐hydroxyaminopurine selectively suppressed melanoma growth in vivo without systemic toxicity. Strikingly, *Staphylococcus epidermidis* colonization protected mice from ultraviolet skin cancer induction. As *Staphylococcus epidermidis* produces multiple bioactive molecules, future studies should explore whether other *Staphylococcus epidermidis*–derived molecules may have anti‐tumoural capacity. It remains unknown also whether some of *Staphylococcus epidermidis* antigens may elicit anti‐cancer activity. Interestingly, as N‐formyl methionine peptides, secreted by *Staphylococcus epidermidis*, enhance CD8+ T lymphocytes,^7^ and increased intra‐tumoural CD8+ T cell infiltration has been correlated with good clinical outcome in melanoma,^83^ it is suggestive that several *Staphylococcus epidermidis‐*derived molecules may serve as promising anti‐cancer drugs. Importantly, future studies also should evaluate how *Staphylococcus epidermidis–*derived 6‐N‐hydroxyaminopurine affects wound healing.

## 3 CLINICAL RELEVANCE

Skin wound repair mouse models try to recreate characteristics of human wound healing after injury. Nevertheless, cutaneous wound healing in mice differ from the one in humans.^84^ Therefore, taking into account the peculiarities of each species is key to correctly interpret the data. Murine and human skin layers differ in thickness and number of cells. The mouse skin is thinner than 25 μm and more loose, while human's is thicker than 100 μm and more adherent to the underlying tissues.^85^, ^86^ Moreover, as mouse epidermis comprises three cell layers, while human's contains 10,^87^ the effect of the microbiota may be transmitted differently in humans. Although experimental manipulations of mouse models in cutaneous microbiota research allow functional and mechanistic investigation on host‐microbe interactions, pitfalls should be considered when translating skin microbiome research results from murine models to humans. Thus, complementary to the study by Linehan et al (2018), it would be interesting to analyse the transcriptome of human derived cutaneous CD8+ T cells in the presence and absence of *Staphylococcus epidermidis*. This study may also contribute to the treatment of chronic wounds, in which the role of the microbiota is still controversial. Some studies demonstrate that, under certain conditions, commensals may participate in chronic infections with negatives effects.^88^ However, little evidence supports the use of systemic antibiotics to promote healing in chronic wounds.^89^ Besides that, as seen recently, the stability of the microbiota is essential in wound healing.^6^ Therapeutic strategies that promote microbiota modulation could lead to improvement of wounds that are difficult to heal. In this context, the use of probiotics has been shown to be beneficial for the treatment of chronic wounds.^90^, ^91^ Chronic wounds may have the presence of multiple microorganisms that could form complex structures, like biofilms.

The bacterial biofilms formation in chronic wounds has been linked to worsening wound healing.^92^ Drugs that prevent biofilm quorum sensing signalling may be potential therapeutic targets.^93^ Nevertheless, the role of such structures in chronic wounds remains unclear.

Multiple challenges in cutaneous microbiome research still need to be addressed for therapy based on specific microbiota targets. How individual microorganisms affect to specific types of immune response could be viewed as a potential source of new therapeutics. In the article by Linehan et al some important aspects that determine more minutely the durability and mechanical details of how *S. epidermidis* modulate immune response, need to be further elucidated. The understanding of atypical functions activated in the immune cells by the modulation of cutaneous microbes may provide therapeutic opportunities.

## 4 CONCLUSION

The study by Linehan and colleagues reveals a new important role of a component of the skin commensal microbiota. Nevertheless, our understanding of cutaneous microbiota biology still remains limited, and future studies should shed light on the complexity and interactions of different cellular components of the cutaneous microenvironment with microbes during wound healing. A great challenge for the future will be to translate the research from mouse models into humans. How human cutaneous microbiota contribute to different stages of wound healing remains to be determined. Improving the availability of human tissue samples will be essential to reach this aim.

## DISCLOSURES

The authors indicate no potential conflicts of interest.

## AUTHOR CONTRIBUTIONS

All authors wrote and commented on the manuscript.
